# Cardiac-Specific Deletion of Pyruvate Dehydrogenase Impairs Glucose Oxidation Rates and Induces Diastolic Dysfunction

**DOI:** 10.3389/fcvm.2018.00017

**Published:** 2018-03-06

**Authors:** Keshav Gopal, Malak Almutairi, Rami Al Batran, Farah Eaton, Manoj Gandhi, John Reyes Ussher

**Affiliations:** ^1^Faculty of Pharmacy and Pharmaceutical Sciences, University of Alberta, Edmonton, AB , Canada; ^2^Alberta Diabetes Institute, University of Alberta, Edmonton, AB, Canada; ^3^Mazankowski Alberta Heart Institute, University of Alberta, Edmonton, AB, Canada

**Keywords:** pyruvate dehydrogenase, glucose oxidation, diabetic cardiomyopathy, cardiac function, diastolic dysfunction

## Abstract

Obesity and type 2 diabetes (T2D) increase the risk for cardiomyopathy, which is the presence of ventricular dysfunction in the absence of underlying coronary artery disease and/or hypertension. As myocardial energy metabolism is altered during obesity/T2D (increased fatty acid oxidation and decreased glucose oxidation), we hypothesized that restricting myocardial glucose oxidation in lean mice devoid of the perturbed metabolic milieu observed in obesity/T2D would produce a cardiomyopathy phenotype, characterized via diastolic dysfunction. We tested our hypothesis via producing mice with a cardiac-specific gene knockout for pyruvate dehydrogenase (PDH, gene name *Pdha1*), the rate-limiting enzyme for glucose oxidation. Cardiac-specific *Pdha1* deficient (*Pdha1*^Cardiac−/−^) mice were generated via crossing a tamoxifen-inducible Cre expressing mouse under the control of the alpha-myosin heavy chain (αMHC-MerCreMer) promoter with a floxed *Pdha1* mouse. Energy metabolism and cardiac function were assessed via isolated working heart perfusions and ultrasound echocardiography, respectively. Tamoxifen administration produced an ~85% reduction in PDH protein expression in *Pdha1*^Cardiac−/−^ mice versus their control littermates, which resulted in a marked reduction in myocardial glucose oxidation and a corresponding increase in palmitate oxidation. This myocardial metabolism profile did not impair systolic function in *Pdha1*^Cardiac−/−^ mice, which had comparable left ventricular ejection fractions and fractional shortenings as their αMHC-MerCreMer control littermates, but did produce diastolic dysfunction as seen via the reduced mitral E/A ratio. Therefore, it does appear that forced restriction of glucose oxidation in the hearts of *Pdha1*^Cardiac−/−^ mice is sufficient to produce a cardiomyopathy-like phenotype, independent of the perturbed metabolic milieu observed in obesity and/or T2D.

## Introduction

The healthy heart is a metabolic omnivore that is dynamically flexible, constantly switching between carbohydrates (i.e., glucose) and fatty acids as its primary fuel source between repeated periods of refeeding and fasting, respectively (1, 2). In the setting of obesity and/or type 2 diabetes (T2D), the heart’s metabolic flexibility dissipates, as the heart increases its reliance on fatty acids as the primary fuel source to meet its oxidative energy requirements (2, 3). For example, studies from Abel and colleagues have demonstrated that both leptin deficient *ob*/*ob* and leptin receptor deficient *db*/*db* mice exhibit robust increases in myocardial fatty acid oxidation rates, which is associated with a marked reduction in glucose oxidation rates (4). Moreover, Larsen and colleagues have observed identical findings in hearts from mice subjected to a sucrose-enriched diet to induce experimental obesity (5). Similarly, short-term high fat feeding of C57BL/6J mice for 2 weeks also produces significant increases in myocardial fatty acid oxidation rates and a corresponding decline in myocardial glucose oxidation rates (6). Such observations have been recapitulated in humans, as PET imaging studies by Peterson et al. have demonstrated a marked increase in fatty acid oxidation rates in the hearts of obese women, which positively correlated with overall insulin resistance (7).

These metabolic observations can be partly explained by the marked increase in circulating free fatty acid and triacylglycerol levels characteristic of obesity/T2D, which increases myocardial fatty acid delivery and subsequent fatty acid oxidation. An increase in myocardial fatty acid oxidation rates leads to a corresponding decrease in glucose oxidation rates through the “Randle Cycle” mechanism, by which fatty acids and glucose compete for oxidative acetyl CoA production (1, 8). In addition, it has also been demonstrated that insulin’s ability to stimulate glucose oxidation is severely diminished in the hearts of animals or humans with obesity and/or T2D (3, 9). However, despite well documented observations regarding the myocardial metabolic phenotype in obesity/T2D, it remains enigmatic as to whether these metabolic perturbations are driving forces behind the pathology of the cardiomyopathy and diastolic dysfunction that characterizes patients with T2D.

Our objective was to determine whether a specific defect in myocardial glucose oxidation was sufficient to reproduce the cardiomyopathy phenotype observed in obesity/T2D, independent of the perturbed metabolic milieu associated with obesity/T2D. Such observations would support the notion that the myocardial metabolic perturbations observed in obesity/T2D are mechanistically involved in the pathology of diabetic cardiomyopathy. As pyruvate dehydrogenase (PDH, gene name *Pdha1*) is the rate-limiting enzyme of glucose oxidation (10), to address our aim we generated and characterized cardiac specific *Pdha1* (*Pdha1*^Cardiac−/−^) mice, investigating potential changes in cardiac function and cardiac glucose/fatty acid oxidation.

## Methods

### Animal Care

All animals received care according to the Canadian Council on Animal Care and all procedures were approved by the University of Alberta Health Sciences Animal Welfare Committee. C57BL/6J wild-type (WT), alpha-myosin heavy chain (αMHC)-MerCreMer (stock no. 005657) and *Pdha1*^flox^ (stock no. 017443) mice were purchased from the Jackson Laboratory, USA. To generate *Pdha1*^Cardiac−/−^ mice, αMHC-MerCreMer transgenic mice expressing tamoxifen-inducible Cre in cardiac myocytes were bred with *Pdha1*^flox^ mice. Cre-induced inactivation of the *Pdha1* gene was carried out via 6 intraperitoneal (i.p.) injections of tamoxifen (50 mg/kg) spread over 8 days in male mice starting at 6–7 weeks of age (11, 12). All mice were allowed five weeks washout post-tamoxifen administration prior to experimentation.

### Western Blotting

Frozen ventricular tissue (20 mg) was homogenized in buffer containing 50 mM Tris HCl (pH 8 at 4°C), 1 mM EDTA, 10% glycerol (wt/vol), 0.02% Brij-35 (wt/vol), 1 mM dithiothreitol, protease and phosphatase inhibitors (Sigma) to prepare myocardial protein extracts as previously described (12, 13). Protein concentration of homogenates was determined via Bradford protein assay kit (Bio-Rad). Samples were resolved via 10% sodium dodecyl sulfate polyacrylamide gel electrophoresis (SDS-PAGE) and transferred onto a 0.45 µm nitrocellulose membrane. Membranes were blocked with 10% fat free milk for 1 h and probed with either anti-PDH (Cell Signaling Technologies) or anti-hsp90 (BD Biosciences) antibodies in 5% fatty acid free bovine serum albumin overnight at 4°C. Immunoblots were visualized with the enhanced chemiluminescence western blot detection kit (Perkin Elmer), visualized with a geldoc imager (Bio-Rad) and quantified with ImageJ software.

### Determination of Plasma Triacylglycerol (TAG), Insulin, and Free Fatty Acid (FFA) Levels

All animals had their food removed for a 2 h period, following which whole-blood was collected from mice via tail bleed. Tail whole-blood was centrifuged at 2,000 × *g* at 4°C for 10 min and the supernatant (plasma) was collected. Plasma TAG (Wako Pure Chemical Industries), insulin levels (ALPCO Diagnostics) and FFA levels (Roche) were quantified using commercially available enzymatic assay kits as per manufacturer instructions.

### Ultrasound Echocardiography

Transthoracic echocardiography ultrasound was performed in 3% isoflurane-anesthetized mice (30–40 MHz; Vevo3100, VisualSonics,Toronto, Canada) to assess left ventricular systolic and diastolic function as previously described (14).

### Isolated Working Heart Perfusions and Assessment of Energy Metabolism

Two weeks post-ultrasound echocardiography analysis, all mice were anaesthetized with i.p. sodium pentobarbital (60 mg/kg), following which the hearts were subsequently excised and immersed in ice-cold Krebs-Henseleit bicarbonate solution. The aorta was then cannulated and equilibrated in the Langendorff mode, following which the left atria was subsequently cannulated and hearts were switched to and perfused in the working mode as previously described (13, 15). Oxygenated Krebs-Henseleit solution consisting of 1.2 mM [9,10-^3^H]palmitate bound to 3% fatty acid free bovine serum albumin and 11 mM [U-^14^C]glucose was delivered to the left atrium at a preload pressure of 15 mmHg, while perfusate was ejected from hearts into the aortic outflow line against a hydrostatic afterload pressure of 50 mmHg. Hearts were perfused aerobically for 40 min in the absence of insulin, and glucose and palmitate oxidation were assessed via measuring ^3^H_2_O and ^14^CO_2_ production as previously described (13, 15). At the end of perfusion, hearts were immediately frozen in liquid N_2_ with Wollenberger tongs pre-cooled to the temperature of liquid N_2_, and stored at −80°C.

### Statistical Analysis

All values are presented as means ± standard error of the mean (SEM). Significant differences were determined by the use of an unpaired, two-tailed Student’s *t*-test, or a one-way ANOVA followed by a Bonferroni post-hoc analysis. Differences were considered significant when *P* < 0.05.

## Results

### Generation of Cardiac-Specific *Pdha1* Deficient Mice

**Figure 1:**
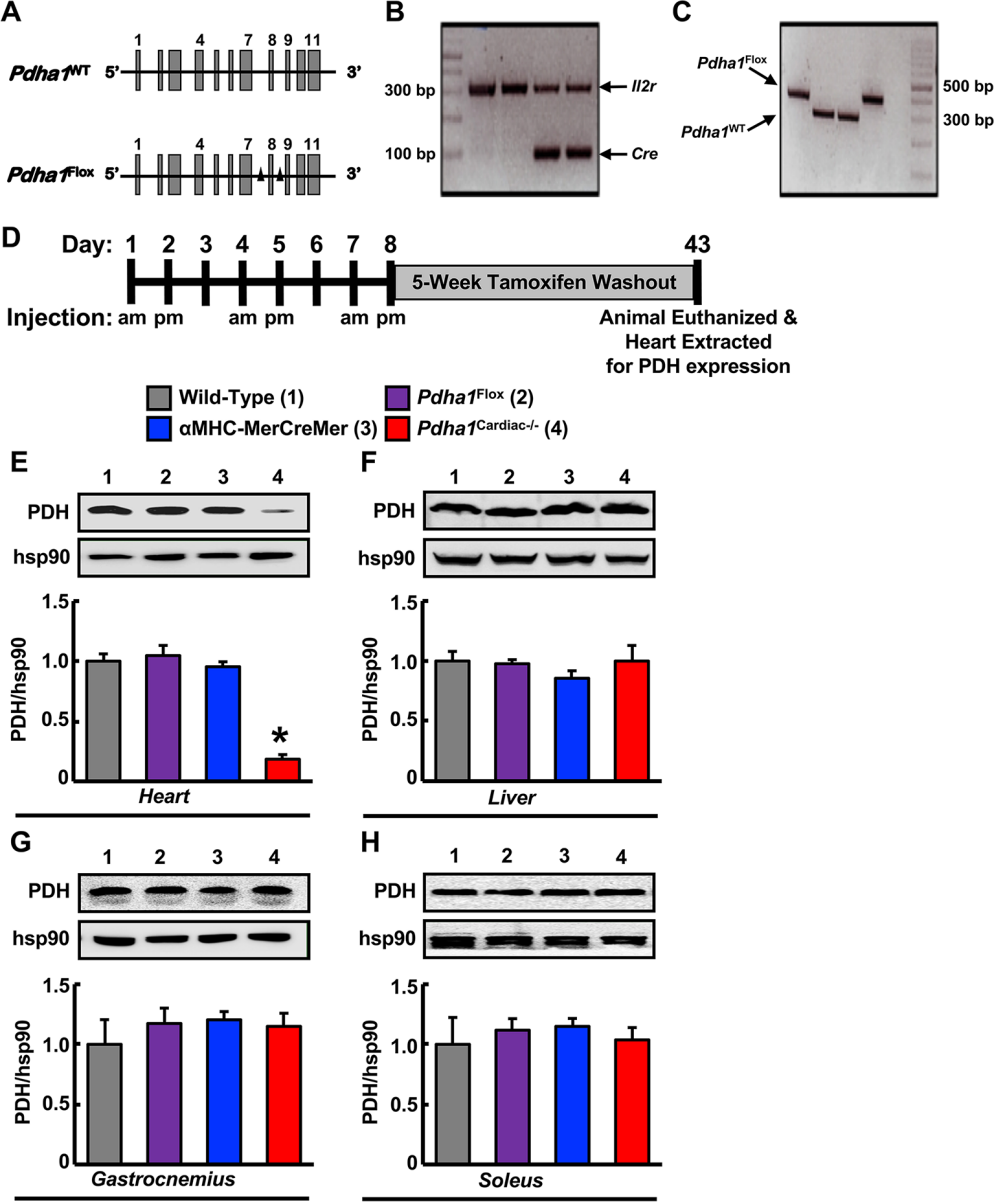
* Generation of cardiac-specific Pdha1-deficient (Pdha1^Cardiac−/−^) mice*. **(A)** Depiction of the *Pdha1* gene (11 exons) in *Pdha1*^WT^ mice and *Pdha1*^Flox^ mice. Black triangles depict the loxP sites flanking exon 8 of the *Pdha1* gene in the *Pdha1*^Flox^ mice. **(B/C)** Cardiac-specific *Pdha1*-deficient mouse was generated by breeding αMHC-MerCreMer transgenic mice expressing a tamoxifen-inducible Cre in cardiac myocytes with *Pdha1*^flox^ mice. PCR genotyping of mouse offspring showing presence of *Cre***(B)** as amplification of a 100 base pair fragment, or presence of a floxed *Pdha1* or wild-type *Pdha1***(C)** gene as amplification of a 380 base pair or 303 base pair fragment, respectively. Amplification of the *Il2r* was utilized as a positive control for PCR amplification. **(D)** Schematic model for induction of cardiac-specific *Pdha1* knockout by 6-injections of tamoxifen indicating whether mice were injected in the morning (am) or late afternoon (pm) of the day. Mice were allowed a 5 week washout period following the last tamoxifen injection prior to experimentation. **(E-H)** Western blot analysis of protein lysates of heart **(E)**, liver **(F)**, gastrocnemius muscle **(G)**, and soleus muscle **(H)** comparing PDH expression in *Pdha1*^Cardiac−/−^ mice (4) versus their various control littermates [Wild-type mice (1), *Pdha1*^Flox^ mice (2), and αMHC-MerCreMer mice (3)] (*n* = 4–7). Values represent mean ± SEM. Differences were determined using 1-way ANOVA followed by a Bonferroni post-hoc analysis. *P < 0.05, significantly different versus all other genotypes.

**Figure 2:**
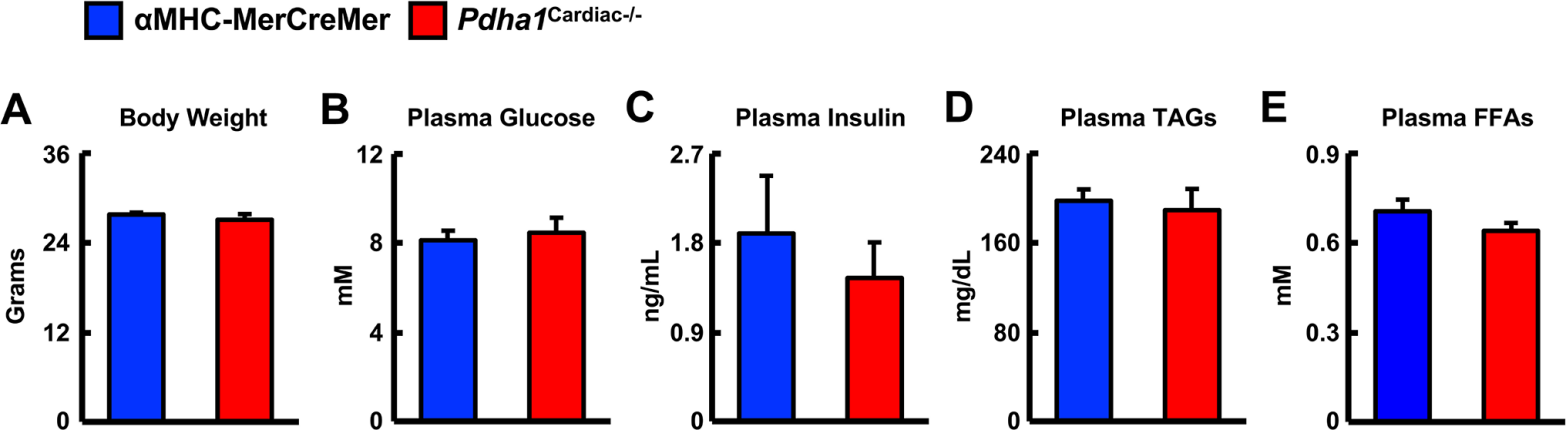
* Baseline parameters in Pdha1^Cardiac−/−^ mice*. **(A)** Body weights, **(B)** plasma glucose levels, **(C)** plasma insulin levels, **(D)** plasma TAG levels, and **(E)** plasma FFA levels in *Pdha1*^Cardiac−/−^ mice and their αMHC-MerCreMer control littermates at 5 weeks post-tamoxifen administration (*n* = 4). Values represent mean ± SEM.

In order to generate *Pdha1*^Cardiac−/−^ mice, floxed *Pdha1* (*Pdha1*^Flox^) mice possessing loxP sites flanking exon 8 of the E1 subunit of *Pdha1* (Figure 1A) were crossed to mice expressing Cre recombinase driven by the cardiac-specific αMHC (αMHC-MerCreMer) promoter (Figure 1B/C). Cre recombinase expression in cardiac myocytes from αMHC-MerCreMer mice is flanked by mutated estrogen receptor ligand-binding domains bound to heat shock protein 90 (hsp90), which can be activated via the selective estrogen receptor antagonist, tamoxifen, but remain insensitive to endogenous estrogen. Thus, we treated WT, *Pdha1*^Flox^, αMHC-MerCreMer, and *Pdha1*^Cardiac−/−^male mice with tamoxifen (50 mg/kg, 6 i.p. injections spread over 8 days) to induce Cre recombinase-mediated excision of loxP flanked DNA regions (Figure 1D). 5 weeks post-tamoxifen treatment, hearts were extracted from all mice with free access to food in the middle of the light cycle for assessment of myocardial PDH expression. As anticipated, we observed an ~85% gene knockdown of *Pdha1* only in myocardial protein extracts from *Pdha1*^Cardiac−/−^ mice, but not from WT, *Pdha1*^Flox^, or αMHC-MerCreMer mice (Figure 1E), similar to what we’ve seen in our previous studies using this approach (11, 12, 16). Importantly, PDH protein expression was similar in the skeletal muscle (gastrocnemius and soleus) and livers of *Pdha1*^Cardiac−/−^ mice and their various control littermates (Figure 1F–H), indicating we had successfully generated a cardiac-specific PDH deficient mouse model. Because cardiac-specific Cre recombinase expression controlled via the αMHC promoter produces a transient cardiomyopathy (17), all subsequent studies were performed using the αMHC-MerCreMer mouse as the control littermate. Assessment of body weight and baseline plasma parameters demonstrated no overt phenotype in *Pdha1*^Cardiac−/−^ mice, as their body weight and circulating glucose, insulin, triacylglycerol, and FFA levels were comparable to their αMHC-MerCreMer control littermates (Figure 2A–E).

### *Pdha1*^Cardiac−/−^ Mice Exhibit Normal Systolic Function with Signs of Diastolic Dysfunction

**Figure 3:**
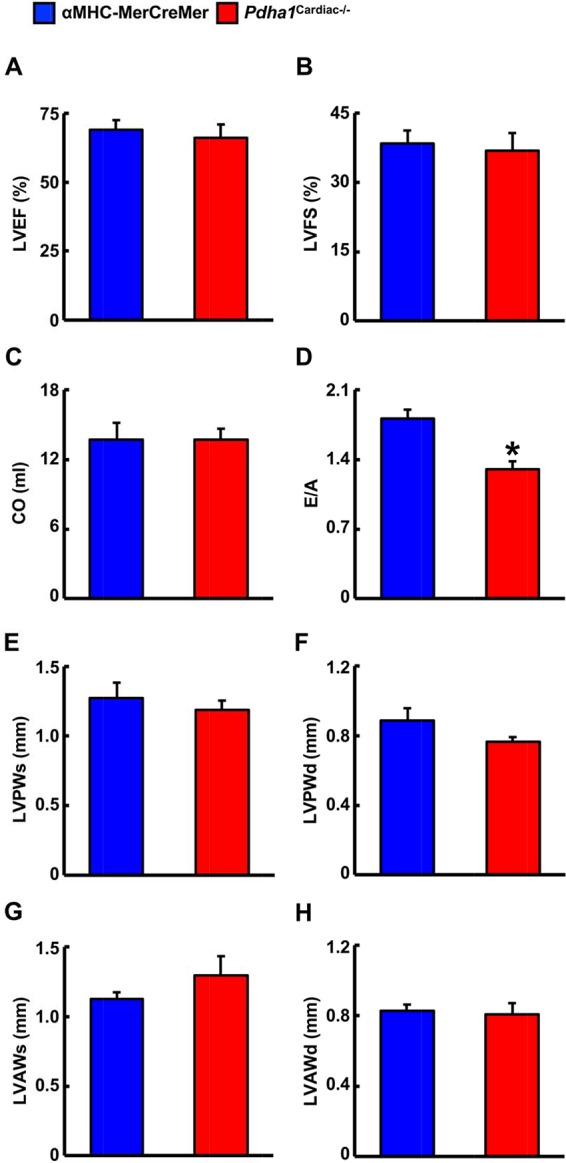
* Pdha1^Cardiac−/−^ mice exhibit normal systolic function with evidence of diastolic dysfunction*. Ultrasound echocardiography was performed in *Pdha1*^Cardiac−/−^ mice and their αMHC-MerCreMer control littermates to assess indices of systolic function; **(A)** left ventricular ejection fraction (LVEF), **(B)** left ventricular fractional shortening (LVFS), and **(C)** cardiac output (CO). **(D)** Diastolic function was assessed via measurement of the mitral E/A ratio. Left ventricular wall dimensions were assessed via measurement of posterior wall thickness during systole (LVPWs), **(F)**, posterior wall thickness during diastole (LVPWd), **(G)**, anterior wall thickness during systole (LVAWs), and **(H)** anterior wall thickness during diastole (LVAWd). Values represent means ± SEM (*n* = 6–7). Differences were determined using an unpaired, two-tailed Student’s *t*-test. *P < 0.05.

We next performed ultrasound echocardiography studies in αMHC-MerCreMer and *Pdha1*^Cardiac−/−^ mice to assess *in vivo* cardiac function. Systolic function appeared normal in* Pdha1*^Cardiac−/−^ mice, since left ventricular (LV) ejection fraction (LVEF), fractional shortening, and cardiac output were similar in *Pdha1*^Cardiac−/−^ mice and their αMHC-MerCreMer control littermates (Figure 3A–C), indicating that impaired myocardial glucose oxidation rates do not adversely affect LV systolic function. Conversely, the mitral E/A ratio was decreased by ~28% in *Pdha1*^Cardiac−/−^ mice (Figure 3D), suggestive of diastolic dysfunction. Regarding cardiac wall dimensions, *Pdha1*^Cardiac−/−^ mice demonstrated no differences in LV posterior and anterior wall thickness in comparison to their αMHC-MerCreMer control littermates (Figure 3E–H).

### *Pdha1*^Cardiac−/−^ Mice Exhibit a Marked Reduction in Glucose Oxidation Rates and a Subsequent Increase in Fatty Acid Oxidation Rates

**Figure 4:**
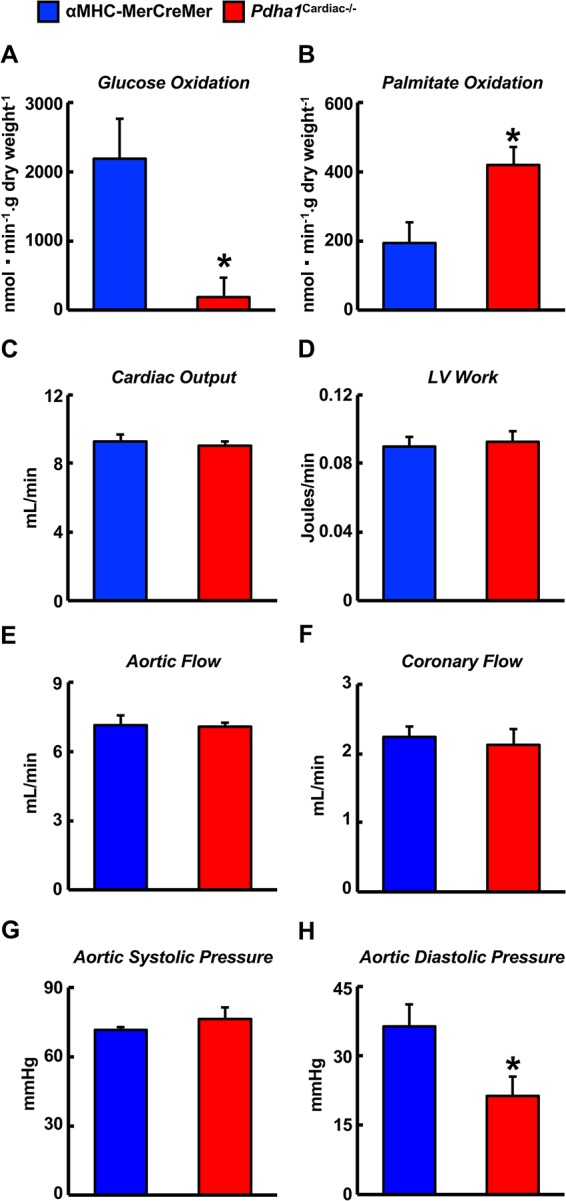
* Altered myocardial energy metabolism and normal ex vivo cardiac function in Pdha1^Cardiac−/−^ mice*. **(A)** Glucose oxidation rates, **(B)** palmitate oxidation rates, **(C)** cardiac output, **(D)** left ventricular (LV) work, **(E)** aortic flow, **(F)** coronary flow, **(G)** aortic systolic pressure, and **(H)** aortic diastolic pressure during isolated aerobic working heart perfusions from *Pdha1*^Cardiac−/−^ and their αMHC-MerCreMer control littermates (*n* = 4, 5). Values represent means ± SEM. Differences were determined using an unpaired, two-tailed Student’s *t*-test. *P < 0.05.

**Figure 5:**
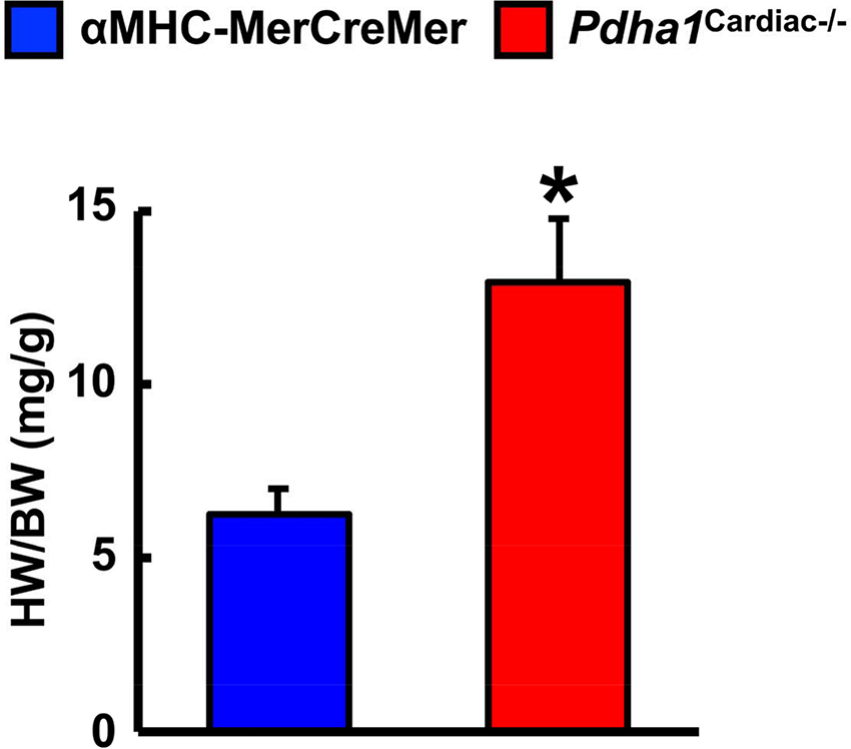
* Pdha1^Cardiac−/−^ mice exhibit cardiac hypertrophy*. Heart weight was normalized to body weight (HW/BW) in *Pdha1*^Cardiac−/−^ mice and their αMHC-MerCreMer control littermates following completion of 40 min aerobic perfusion. Values represent means ± SEM (*n* = 5). Differences were determined using an unpaired, two-tailed Student’s *t*-test. *P < 0.05.

Two weeks following the assessment of *in vivo* cardiac function via ultrasound echocardiography, all mice were subjected to isolated working heart perfusion studies for the assessment of cardiac glucose and fatty acid oxidation rates during hrs 4 through 8 of their light cycle. Consistent with a cardiac-specific deficiency of PDH, *Pdha1*^Cardiac−/−^ mice exhibited a marked reduction in glucose oxidation rates following aerobic perfusion in the isolated working mode (Figure 4A). In contrast, fatty acid oxidation rates were increased in working hearts from *Pdha1*^Cardiac−/−^ mice (Figure 4B), likely due to a “Randle Cycle” effect (1, 8). Importantly, the altered metabolic profile in hearts from *Pdha1*^Cardiac−/−^ mice did not adversely affect their *ex vivo* cardiac function, as both cardiac output and left ventricular work were similar in *Pdha1*^Cardiac−/−^ mice and their αMHC-MerCreMer control littermates (Figure 4C/D). Moreover, there were no differences in aortic and coronary flows, or aortic systolic pressure during isolated working heart perfusions from *Pdha1*^Cardiac−/−^ mice and their αMHC-MerCreMer control littermates (Figure 4E–G), though aortic diastolic pressure was significantly decreased in *Pdha1*^Cardiac−/−^ mice (Figure 4H). Interestingly, measurement of heart weight/body weight ratios upon completion of the 40 min aerobic perfusion protocol, revealed substantial cardiac hypertrophy in *Pdha1*^Cardiac−/−^ mice (Figure 5).

## Discussion

Despite being a major fuel source for the heart, our observations demonstrate that a marked reduction in glucose oxidation in the heart secondary to cardiac-specific deletion of PDH does not adversely affect LV systolic function. This is likely due to the corresponding increase in fatty acid oxidation, ensuring that the oxidative energy needs of the heart are being met. On the contrary, our study provides support for the concept that a specific defect in myocardial glucose oxidation is sufficient to produce a diabetic cardiomyopathy-like phenotype, as *Pdha1*^Cardiac−/−^ mice exhibited signs of diastolic dysfunction, reflected by their decreased mitral E/A ratio in comparison to their αMHC-MerCreMer control littermates.

Numerous preclinical studies have demonstrated that obesity and/or diabetes severely alters myocardial energy metabolism, such that the myocardium is heavily dependent on fatty acid oxidation to meet its energy requirements, often at the expense of declining glucose oxidation rates (1, 3). Indeed, myocardial metabolism in obese leptin receptor-deficient *db*/*db* mice is strikingly similar to *Pdha1*^Cardiac−/−^ mice, as isolated working heart perfusion studies have demonstrated marked increases in fatty acid oxidation rates and decreases in glucose oxidation rates (4, 18). Moreover, *db*/*db* mice exhibit a decline in the mitral E/A ratio that is comparable to what we observed in *Pdha1*^Cardiac−/−^ mice (19). Similarly, experimental obesity due to chronic supplementation with a high-fat diet also results in marked increases and decreases in fatty acid oxidation and glucose oxidation, respectively, during isolated working heart perfusion experiments, whereas insulin-stimulated glucose oxidation is heavily diminished (6, 9). These preclinical findings appear to translate to the clinical scenario, as PET imaging studies in either obese women or men with T2D demonstrate marked increases in myocardial fatty acid oxidation rates and a decline in glucose utilization (7, 20). Intriguingly, therapeutic interventions that restore glucose oxidation rates in the hearts of mice subjected to experimental models of obesity and/or T2D, either directly or secondary to an inhibition of fatty acid oxidation, result in a mitigation of the ensuing cardiomyopathy (9, 14, 21). Likewise, this has also been seen in experimental models of type 1 diabetes, as pharmacological activation of PDH via treatment with dichloroacetate, augmented contractile function in isolated working hearts from rats treated with streptozotocin (22).

Recent studies also suggest that glucose oxidation is perturbed during diastolic dysfunction due to chronic infusion with angiotensin II (23), whereas treatment with an angiotensin II type 1 receptor antagonist (irbesartan) reverses experimental diastolic dysfunction, which is associated with a restoration of myocardial glucose oxidation rates (23). Of interest, mice harboring a whole-body deficiency of PDH kinase 4 (*Pdk4*^−/−^), a key enzyme that inactivates PDH/glucose oxidation, were protected against angiotensin II-induced diastolic dysfunction (24). Furthermore, isolated working heart perfusion studies demonstrated that glucose oxidation rates were increased in angiotensin II-infused *Pdk4*^−/−^ mice versus their wild-type littermates (24).

**Figure 6:**
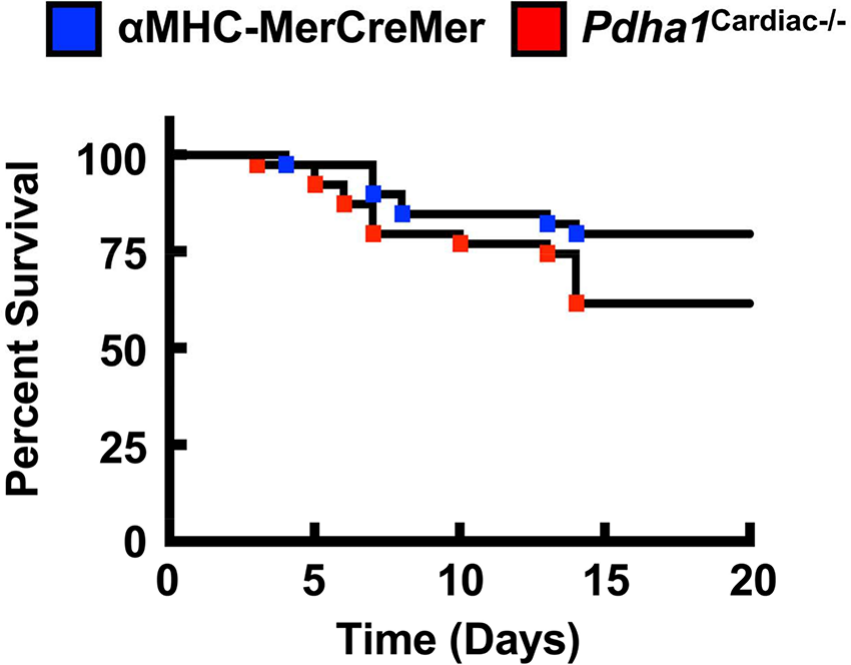
* Pdha1^Cardiac−/−^ mice exhibit increased sensitivity to Cre recombinase-related mortality due to fatal cardiomyopathy*. Survival rates in *Pdha1*^Cardiac−/−^ mice and their αMHC-MerCreMer control littermates starting from the day of first tamoxifen injection (*n* = 39). Differences were determined using a Kaplan-Meier survival curve analysis. *P < 0.05.

Our findings share some similarities but also some differences from a previous study that generated a *Pdha1*^Cardiac−/−^ mouse model (25). While Sun and colleagues also observed a marked reduction in glucose oxidation rates as expected for a mouse heart deficient in PDH, they did not observe a corresponding increase in fatty acid oxidation rates. This may explain why their *Pdha1*^Cardiac−/−^ mice exhibited premature mortality, as evidenced by no *Pdha1*^Cardiac−/−^ mice surviving beyond 16 weeks of age in this particular study, which could be due to energetically compromised hearts and death via heart failure. The *Pdha1*^Cardiac−/−^ mice studied by Sun and colleagues did not demonstrate significant reductions in systolic function at 1 month post-tamoxifen administration, similar to what we observed in our *Pdha1*^Cardiac−/−^ mice at 5 weeks post-tamoxifen administration. Nevertheless, at 2 months post-tamoxifen administration, their *Pdha1*^Cardiac−/−^ mice showed signs of heart failure with LVEFs <30%. Thus, it is possible that if we allowed our mice to age longer that perhaps we would have also observed systolic dysfunction in our *Pdha1*^Cardiac−/−^ mice. Moreover, another aspect worth considering is that the method of tamoxifen-mediated gene deletion via Cre recombinase was not identical between our study and that of Sun et al. We administered tamoxifen to our mice 6 times at a dose of 50 mg/kg body weight spread over 8 days (Figure 1D), whereas Sun and colleagues administered tamoxifen for 5 consecutive days to their mice at a dose of 80 mg/kg body weight. Since Cre-recombinase activity in cardiac myocytes can produce a potentially fatal cardiomyopathy in αMHC-MerCreMer mice (17), it is possible that the severity of the Cre-recombinase-induced cardiomyopathy was exacerbated by cardiac-specific deletion of *Pdha1*, explaining the accelerated mortality in *Pdha1*^Cardiac−/−^ mice from Sun et al. (25). In support of this notion, our *Pdha1*^Cardiac−/−^ mice were more sensitive to the Cre recombinase mediated cardiomyopathy/mortality during tamoxifen treatment when compared to their αMHC-MerCreMer control littermates (Figure 6). However, all our *Pdha1*^Cardiac−/−^ mice that survived the tamoxifen treatment protocol were able to survive until study completion. Interestingly, while ultrasound echocardiography revealed no gross LV structural abnormalities in hearts from *Pdha1*^Cardiac−/−^ mice, when we perfused our mice for assessment of cardiac energy metabolism at 2 weeks post-ultrasound, *Pdha1*^Cardiac−/−^ mice now demonstrated a marked cardiac hypertrophy, similar to that reported by Sun and colleagues (25). Likewise, Sun et al. also observed indices of impaired diastolic function in their *Pdha1*^Cardiac−/−^ mice, as reflected by elevations in left ventricular interior diameter during diastole.

Despite the plethora of evidence showing a strong association between diabetic cardiomyopathy and impaired myocardial glucose oxidation, our study demonstrates that extinguishing glucose oxidation via cardiac-specific deletion of PDH is sufficient to produce a cardiomyopathy-like phenotype characterized via diastolic dysfunction and cardiac hypertrophy. Importantly, our studies were done in lean animals in the absence of the perturbed metabolic milieu associated with obesity and/or T2D, indicating that a reduction in myocardial glucose oxidation rates *per se* is likely a contributing factor to the pathology of obesity- and/or diabetes-related cardiomyopathy. Taken together, it would suggest that pharmacological development of PDH agonists to stimulate glucose oxidation (e.g., dichloroacetate) may be a novel therapeutic approach to attenuate diabetic cardiomyopathy, and the generation of *Pdha1*^Cardiac−/−^ mice will prove to be a valuable tool in confirming the validity of such a strategy.

## Ethics

This study was carried out in accordance with the recommendations of the Canadian Council on Animal Care. All animal protocols were approved by the University of Alberta Health Sciences Animal Welfare Committee.

## Author Contributions

KG, MA, RB, FE and MG performed the research. KG and JU designed the research study. KG and MA analyzed the data. KG and JU wrote the paper. KG, MA, RB, and JU edited and revised the discussion. All authors approved the final version of the manuscript.

## Conflict of Interest Statement

The authors declare that the research was conducted in the absence of any commercial or financial relationships that could be construed as a potential conflict of interest.
